# Amylase-Producing Lung Cancer with a Positive Epidermal Growth Factor Receptor Mutation Treated With Gefitinib: A Case Report

**DOI:** 10.14740/wjon778w

**Published:** 2014-03-11

**Authors:** Seigo Minami, Ryu Jokoji, Suguru Yamamoto, Yoshitaka Ogata, Taro Koba, Shinji Futami, Yu Nishijima, Moto Yaga, Kentaro Masuhiro, Masahiko Tsujimoto, Kiyoshi Komuta

**Affiliations:** a Department of Respiratory Medicine, Osaka Police Hospital, Osaka, Japan; b Department of Pathology, Osaka Police Hospital, Osaka, Japan; c Seigo Minami and Ryu Jokoji contributed equally to this report

**Keywords:** Salivary-type amylase, Epidermal growth factor receptor mutation, Adenocarcinoma, Lung cancer, Gefitinib

## Abstract

A 60-year-old woman was diagnosed with metastatic pulmonary adenocarcinoma (c-stage IV) with an L858R point mutation in the gene encoding epidermal growth factor receptor (EGFR). Serum amylase levels were elevated (1,531 IU/L) with the salivary-type enzyme dominating. First-line chemotherapy using carboplatin plus paclitaxel reduced serum amylase levels, although second-line gefitinib eventually failed to control tumor growth and hyperamylasemia after 4.5 months of treatment. The cancer cells harbored a positive EGFR mutation and secreted amylase. The number of amylase-producing cancer cells and the immunochemical staining intensity for amylase were significantly reduced after gefitinib treatment. This was a rare case of a lung cancer that expressed amylase and harbored a positive EGFR mutation.

## Introduction

Since Weiss et al presented the first case of a bronchogenic carcinoma associated with elevated serum amylase levels in 1951 [1], several similar cases have been documented [2-6]. The incidence of this type of lung cancer remains unknown, although a very old study reported only 1-3% of patients with lung carcinoma [7]. The major histological subtype and clinical characteristics of these cases mainly comprise adenocarcinoma and the secretion of salivary-type amylase.

Activation of the somatic mutations of the gene encoding epidermal growth factor receptor (EGFR) is the major determinant of the clinical response to EGFR tyrosine kinase inhibitors (TKIs), such as gefitinib and erlotinib, in patients with non-small cell lung cancer [8, 9]. More than 90% of these mutations occur in exons 19-21, which encode the protein tyrosine kinase domain of the receptor [10]. These mutations are more frequently detected in adenocarcinoma (30%) than in non-adenocarcinoma (2%) [10].

Here, we report on a patient with an amylase-producing lung adenocarcinoma with an activating EGFR mutation.

## Case Report

**Figure 1:**
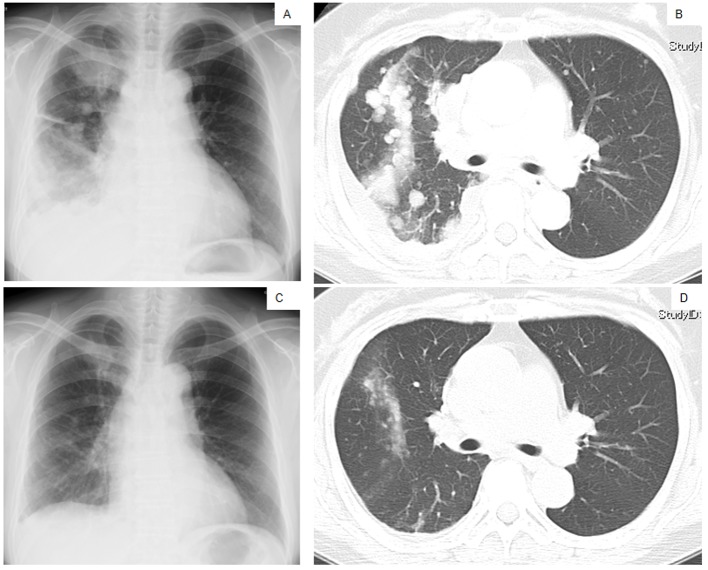
Chest X-ray films (A, C) and a CT scan of the middle lung fields (B, D) taken in April 2010 before first-line chemotherapy using carboplatin plus weekly paclitaxel (A, B), and taken in May 2010 after the second course of the first-line chemotherapy (C, D).

A 60-year-old Japanese woman who had never smoked was referred to our hospital in March 2010 because of a persistent cough and left subclavicular lymphadenopathy. A chest X-ray (Fig. 1A) and a computed tomography (CT) scan (Fig. 1B) showed multiple nodules in both lungs. The patient did not present with any neurological symptoms, although an enhanced brain CT scan revealed multiple metastatic nodules in the brain. According to the results of a cytological examination of a bronchial lavage and radiographic investigations, she was diagnosed with pulmonary adenocarcinoma (cT_4_N_3_M_1_, stage IV). The serum amylase level was elevated (1,531 IU/L; normal level ≤ 106 IU/L), and amylase isozyme patterns identified the salivary type as the most abundant (94%). The pancreas and salivary glands were unlikely to have had any clinical involvement in the development of hyperamylasemia. At that time, we failed to immunohistochemically demonstrate amylase production in cancer tissue because of small number of cancer cells in the bronchoscopical sample. The level of carcinogenic embryonic antigen (CEA) was within the normal range. An EGFR L858R point mutation was detected in exon 21 from the bronchial lavage specimen using the peptide nucleic acid-locked nucleic acid polymerase chain reaction (PNA-LNA PCR) clamp method.

**Figure 2:**
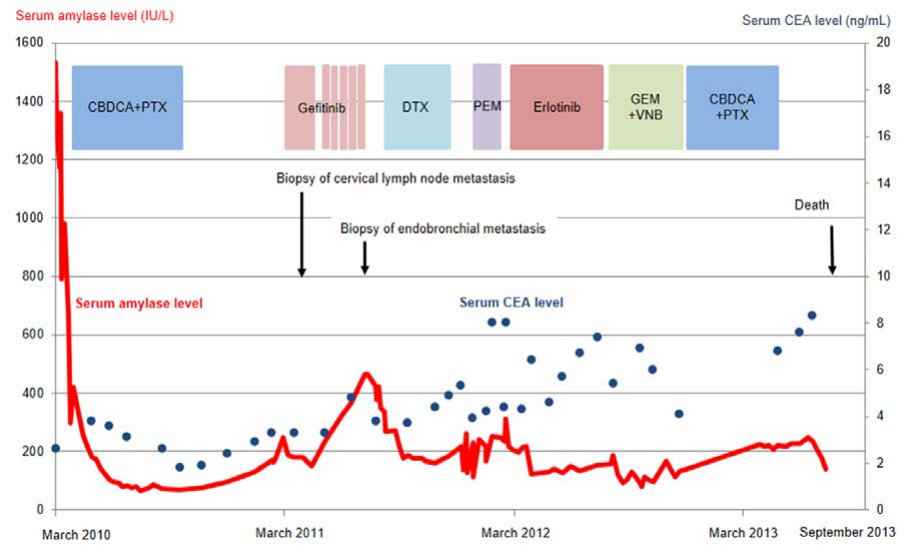
Serum amylase (red line) and CEA (blue dots) levels as a function of chemotherapy regimens. CBDCA, carboplatin; PTX, paclitaxel; DTX, docetaxel; PEM, pemetrexed; GEM, gemcitabine; VNB, vinorelbine; CEA, carcinogenic embryonic antigen.

**Figure 3:**
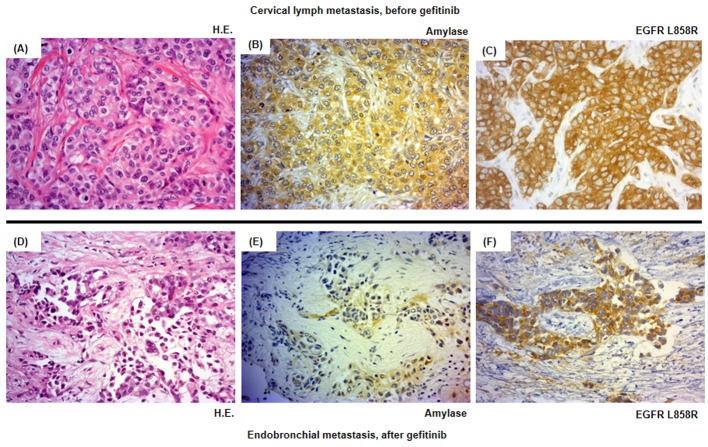
Histological analysis of the patient’s tumor cells. Magnification × 500. (A-C) Cervical lymph metastasis resected in March 2011 before the introduction of the second-line gefitinib treatment. (D-F) Endotracheal metastasis in August 2011 after progressive disease following gefitinib treatment. (A, D) Hematoxylin and eosin staining (HE staining). (B, E) Salivary-type amylase staining. (C, F) EGFR (L858R mutation-specific) staining.

The patient was treated with a combination of carboplatin (area under the blood concentration time curve, 6 mg/min/mL) plus weekly paclitaxel (70 mg/m^2^) as the first-line regimen. Her amylase levels rapidly decreased after the introduction of chemotherapy and eventually reached the normal range (Fig. 2). Chest X-ray (Fig. 1C) and CT scan (Fig. 1D) results showed a good partial response to chemotherapy. In March 2011, at 6 months after the last administration of the first-line regimen, multiple metastatic lesions in the lung and brain had increased in size and progression was documented; however, the serum amylase level remained slightly elevated (247 IU/L) (Fig. 2). At the same time, we performed a biopsy of a newly enlarged cervical lymph node metastasis. Immunohistochemical analysis detected anti-salivary type amylase using an antibody against amylase (sheep polyclonal antibody, #BGS0480-0404; Biogenesis, Poole, UK) (Fig. 3B), and the L858R mutant of EGFR was detected using a specific cognate antibody (clone 43B2 rabbit monoclonal antibody; Cell Signaling Technology, Massachusetts, USA) [11] (Fig. 3C).

**Figure 4:**
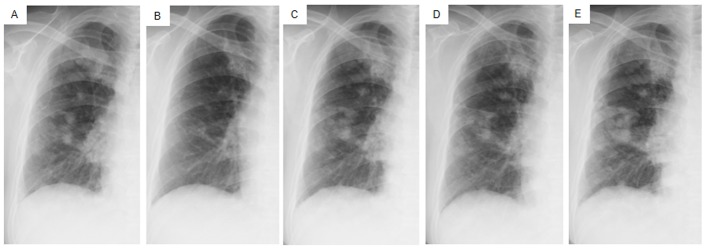
Chest X-ray films of the right lung field taken late in March 2011 before the administration of gefitinib (A), taken early in May at 1.5 months after initiating gefitinib and just before the suspension of gefitinib due to grade 3 elevation of alanine aminotransferase levels (B), taken early in June at the resumption of gefitinib on alternative days (C), taken in mid-July at the 1.5 months after the resumption of gefitinib (D), taken in mid-August after the documentation of progressive disease and 4 days after discontinuation of gefitinib (E).

Daily treatment with gefitinib (250 mg/day) as the second-line regimen induced a remarkable tumor reduction seen on chest X-ray film within the initial 1.5 months of gefitinib treatment (Fig. 4A, B), but treatment was suspended for 3 weeks because of grade 3 elevation of alanine aminotransferase levels. Gefitinib treatment was resumed on alternate days because of recovery of abnormal hepatic function and rapid progression of lung tumor on chest X-ray (Fig. 4C). However, alternative day administration of gefitinib did not respond (Fig. 4D). We eventually documented progressive disease (PD) 4.5 months later (Fig. 4E). During this period, serum amylase levels initially decreased, but gradually increased up to 465 IU/L after gefitinib treatment was suspended (Fig. 2). Amylase isozyme patterns kept the salivary type as the most abundant (79% in March 2011, at the beginning of gefitinib, and 93% in mid-August 2011, 6 days after the discontinuation of gefitinib). In August 2011, after documentation of PD following gefitinib treatment, we obtained a bronchoscopical specimen of a new endotracheal metastasis, while neither bronchoscopical biopsy of intrapulmonary metastases nor surgically resected biopsy of a slightly enlarged cervical lymph node harvested cancerous tissue. PNA-LNA PCR clamp analysis of the endotracheal specimen revealed that the EGFR L858R point mutation was present; however, secondary EGFR mutations such as EGFR T790M were not detected. Furthermore, staining was negative to slightly positive and scattered when the sections were reacted with the anti-salivary type amylase antibody (Fig. 3E), and immunohistochemical analysis using the mutant-specific antibody detected positive expression of the EGFR L858R mutant (Fig. 3F). Docetaxel, pemetrexed and erlotinib were used as the third-, fourth- and fifth-line regimens and a second trial of carboplatin plus paclitaxel was used as the sixth-line regimen. Erlotinib, another EGFR-TKI, only maintained stable disease for 6 months. The patient died of lung cancer in September 2013 at 3.5 years after referral.

## Discussion

Although several cases of amylase-producing lung cancer have been reported, there has been only one case report regarding lung cancer with hyperamylasemia and a positive EGFR mutation. Ko et al described a patient who had lung adenocarcinoma and hyperamylasemia with an in-frame deletion of EGFR exon 19, and achieved a good response to gefitinib; however, this study only detected hyperamylasemia, but did not demonstrate amylase production in the lung cancer cells [12]. In contrast, our immunohistochemical analysis of serial sections of cancer tissue that had been obtained before the administration of gefitinib revealed diffuse expression of both amylase and a mutated form of EGFR. This finding ruled out the coexistence of two kinds of populations of cancer cells that produced amylase or harbored activating EGFR mutations, but demonstrated the existence of cancer cells that expressed amylase and harbored an activating EGFR mutation.

The clinical usefulness of hyperamylasemia as a tumor marker is controversial. The amylase levels detected in tumor tissues of some patients did not correlate with hyperamylasemia [6, 13]; however, serum amylase levels served as a clinically useful tumor marker for other patients [14, 15]. In our case, amylase levels reflected tumor status and were more useful than CEA, conventional tumor markers, at least during first- and second-line chemotherapies.

Despite practical difficulties in performing a second lung cancer biopsy, we fortunately obtained sufficient cancer tissue before and after gefitinib treatment. The metastatic cervical lymph node obtained before gefitinib treatment stained positively for both amylase and a mutated form of EGFR, while the endotracheal metastatic polyp showed positive staining for a mutated form of EGFR after gefitinib treatment, but negative to slightly positive for amylase. Thus, staining intensities for amylase were remarkably reduced before and after gefitinib treatment, despite the difficulty in comparison of the different sites of biopsy. In contrast to reduction of staining intensities for amylase, serum amylase level was increasing gradually during gefitinib treatment. Serum amylase levels correlated with tumor activity through the clinical course during the first- and second-line chemotherapies. The discrepancy between immunohistochemical staining and serum levels of amylase can be regarded as the result of continuous response of EGFR-TKI even beyond PD, because EGFR-TKI in general, gradually develops resistance. We doubted another mechanism other than amylase production in the lung cancer cells because of the following two reasons. First, both at the start of gefitinib treatment, May 2011, and at the documented progression, August 2011, we did not detect any metastatic lesions in the pancreas and salivary glands and also confirmed the dominant secretion of salivary-type amylase in the serum. Second, despite of tiny bronchoscopical samples, we did not detect anywhere other than cancer cells stained by amylase. Thus, we considered that salivary-type amylase was produced exclusively by the cancer cells throughout the clinical course.

Relationship between amylase secretion and EGFR downstream signaling is unknown. Amylase secretion from experimental rat pancreatic acinar cells was regulated through EGFR downstream signaling via the inositol triphosphate pathway and was stimulated by a high concentration of EGF [16]. However, this scenario has not been reported from lung cancer. Moreover, unlike our case, mutations of the EGFR gene were not detected in a human amylase-producing lung adenocarcinoma cell line [17]. Thus, an accidental coexistence of these two characteristics was likely in our cancer cells.

There were several limitations regarding our case study. First, biopsy sites differed before and after gefitinib treatment. We only detected amylase-producing cancer cells with an activating EGFR mutation before gefitinib treatment, and there were cancer cells that produced less amylase but still maintained an activating EGFR mutation after gefitinib treatment. We could not directly demonstrate that the cancer cells reduced or lost their own amylase production during the first- and second-line chemotherapies. Second, our immunohistochemical study did not quantitatively measure amylase production levels in cancer tissues. Although the endotracheal metastatic specimen suggested reduced or lost expression of amylase in cancer cells after gefitinib treatment, we could not directly compare amylase production levels between the different metastatic specimens before and after gefitinib treatment. Thus, we have to be careful in interpreting the immunohistochemical differences before and after gefitinib treatment.

In conclusion, we have presented a rare case of lung adenocarcinoma that produced amylase and harbored an EGFR-activating mutation. A detailed trace of cancer cells with two unique propensities may help clarify the mechanisms of recurrence or acquired drug resistance in cancer cells.
